# Non-thermal plasma enhances growth and salinity tolerance of bok choy (*Brassica rapa* subsp. *chinensis*) in hydroponic culture

**DOI:** 10.3389/fpls.2024.1445791

**Published:** 2024-09-23

**Authors:** Mayura Veerana, Wirinthip Ketya, Eun-Ha Choi, Gyungsoon Park

**Affiliations:** ^1^ Department of Applied Radiation and Isotopes, Faculty of Science, Kasetsart University, Bangkok, Thailand; ^2^ Plasma Bioscience Research Center, Department of Plasma-Bio Display, Kwangwoon University, Seoul, Republic of Korea; ^3^ Department of Electrical and Biological Physics, Kwangwoon University, Seoul, Republic of Korea

**Keywords:** *Brassica rapa* subsp. *chinensis*, atmospheric pressure non-thermal plasma, hydroponic culture, plasma-treated solution, plant growth and development, reactive oxygen and nitrogen species, salinity stress

## Abstract

In this study, we aimed to examine the growth, physiological and biochemical status, and responses to salinity stress of bok choy (*Brassica rapa* subsp. *chinensis*) cultivated in a hydroponic system with a plasma-treated solution. Plasma gas generated using a cylindrical dielectric barrier discharge or air (control) was injected into Hoagland nutrient solution once a week for different durations (0, 5, and 10 min). After 4 weeks, the length of the shoots and roots, number of leaves, and dry weight of bok choy plants significantly increased in individuals grown with Hoagland solution treated with plasma gas for 10 min. An increase in dry weight of individual plants of approximately 80.5% was observed in plants in the plasma-treated group compared to those in a control group. The levels of chlorophyll, total soluble proteins, and nitrogen uptake, and transcription of genes related to salinity stress tolerance—*WRKY2*, *HHP3*, and *ABI1*— were also significantly elevated in bok choy grown with plasma treated Hoagland solution. Moreover, when exposed to 20 mM NaCl, plant length and leaf number were significantly increased, in the group grown with Hoagland solution treated with plasma gas for 10 min. Level of H_2_O_2_ was significantly elevated in the treated nutrient solutions. In plants grown with the treated nutrient solution, intracellular NO was highly detected in the cell division and elongation zone of roots. Our findings suggest that plasma treatment of nutrient solutions in hydroponic culture systems may improve the growth, physiological and biochemical status, and tolerance to salinity stress in plants, and a crucial role of H_2_O_2_ generated in the treated nutrient solutions may play in this improvement.

## 1 Introduction

The increase in population size together with climate change are major obstacles to improving the food security index. The global population is experiencing a steady increase, projected to reach 8.5 billion people by 2030, 9.7 billion by 2050, and 10.9 billion by 2100 (Desa, 2019). Simultaneously, productivity in the agricultural sector has been declining, impacted by the effects of climate change (Kumar et al., 2022). Crop production is affected by various physical, biological, and anthropogenic stressors, which can reduce crop yields by 25–50% (Savary et al., 2012). Traditional agricultural practices cannot currently satisfy the demand for a sustainable food supply of the global population. Therefore, the demand for alternative strategies to overcome these difficulties has grown. Indoor agriculture and the enhancement of plant tolerance to environmental stresses have emerged as potential alternatives to increase production, with intensive research conducted in both fields (Nguyen et al., 2018; Mempel et al., 2021). Hydroponic culture systems represent a major and promising technology applied in indoor agriculture, owing to its usefulness and economic value (Hati and Singh, 2021). These have been technologically upgraded through the integration of smart technology, sanitation tools, and LED technology (Fuentes-Peñailillo et al., 2024). The promotion of plant tolerance to environmental stresses has been extensively studied to identify strategies that can respond to climate change (Redman et al., 2011; Wu et al., 2018; Dahal et al., 2019; Fiodor et al., 2021; Ullah et al., 2021). Developing innovative methods to increase the efficiency of hydroponic cultures and plant tolerance to environmental stresses is required, while minimizing potential negative effects of agricultural activities on the environment.

Plasma is a potential tool that may be applied in hydroponic culture systems to improve plant vitality and productivity. Plasma is an ionized gas, often referred to as the fourth state of matter (Langmuir, 1929; Tendero et al., 2006). It can be created by applying high voltage to gas, causing atoms to ionize and produce chemically reactive components, such as electrons, positive and negative ions, UV photons, free radicals, and resting or excited atoms (Langmuir, 1929). Generally, plasma is classified into thermal plasma (hot) and non-thermal plasma (cold) based on the temperature of its electrons and ions (Tendero et al., 2006; Woedtke et al., 2013). Non-thermal plasma generated at atmospheric pressure has garnered recognition as a cutting-edge, eco-friendly technology that may be applied in the agricultural industry to enhance plant performance and sustainability (Gao et al., 2022). Direct treatment with atmospheric pressure non-thermal plasma has been shown to improve seed germination, plant growth, and reproduction in studies (Serr et al., 2021; Waskow et al., 2021; Fathi, 2022; Sedhai et al., 2023; Guragain et al., 2024; Motrescu et al., 2024); most have shown that plasma promotes seed germination and plant growth by breaking the seed coat and allowing a more effective penetration of water and nutrients. Furthermore, it stimulates the production of enzymes, hormones, and growth factors that promote seedling growth, resulting in faster and more uniform germination and, ultimately, greater crop yields (Adhikari et al., 2020; Song et al., 2020). In addition to direct treatment, indirect treatment can be conducted by using plasma-activated water (PAW) or solutions that are made by exposing plasma to water or solutions to generate with reactive oxygen and nitrogen species, including OH•, O•, H•, ONOO^−^, NO•, and H_2_O_2_ (Kaushik et al., 2019; Zhou et al., 2020; Shaji et al., 2023). Improvements in seed germination and plant growth using PAW have been achieved in various vegetables and crops (for review Gao et al., 2022), such as corn (Lamichhane et al., 2021), radish sprout (Iwata et al., 2019), spinach (Kang et al., 2019), pea (Gao et al., 2019), and tomato (Adhikari et al., 2019). The increase in growth and development in plants treated with PAW is closely associated with the synergistic action of aqueous nitrite (NO_2_^−^), nitrate (NO_3_^−^), and ammonium ions (NH_4_^+^), as well as hydrogen peroxide (H_2_O_2_) species (Zhang et al., 2017; Judée et al., 2018), which activate plant growth regulators, alter levels of plant hormones, and induce stress tolerance responses (Adhikari et al., 2020; Konchekov et al., 2023). In addition, plasma can enhance nutrient availability and absorption by changing the physicochemical characteristics of the soil, decreasing the need for fertilizers and improving nutrient-use efficiency (Konchekov et al., 2023). Other studies have further shown that the effectiveness of direct and indirect non-thermal plasma treatments on plants depends on several factors, such as plant species, plasma source, voltage, pressure, feeding gases, gas flow rate, treatment time, plasma gap distance, moisture content, and type of liquid solution (Kaushik et al., 2019) (Adhikari et al., 2020).

The production of one of the most important vegetables in Asia—*Brassica rapa* subsp. *chinensis*, commonly known as bok choy or pak choi—is adversely affected by a variety of biotic and abiotic stresses (Zhang et al., 2014; Kayum et al., 2015). Currently, most applications of atmospheric pressure non-thermal plasma and PAW in agriculture focus on seeds and plants growing in soil, with relatively little research conducted on hydroponic plant cultivation (Carmassi et al., 2022) (Ruamrungsri et al., 2023) (Date et al., 2023). The goal of this study was to investigate the growth, physiological and biochemical status, and tolerance to salinity stress in bok choy plants cultured in a hydroponic system with a nutrient solution treated with gas generated using cylindrical dielectric barrier discharge (DBD) plasma. The results of our investigation suggest that plasma treatment of nutrient solution in hydroponic culture system can positively impact the growth, physiological and biochemical status, as well as the salinity stress tolerance of plants. Furthermore, our research has highlighted the significant role that H_2_O_2_, which is generated in the treated nutrient solutions, may play in contributing to these positive outcomes.

## 2 Materials and methods

### 2.1 Hydroponic culture and plasma treatment

**Figure 1:**
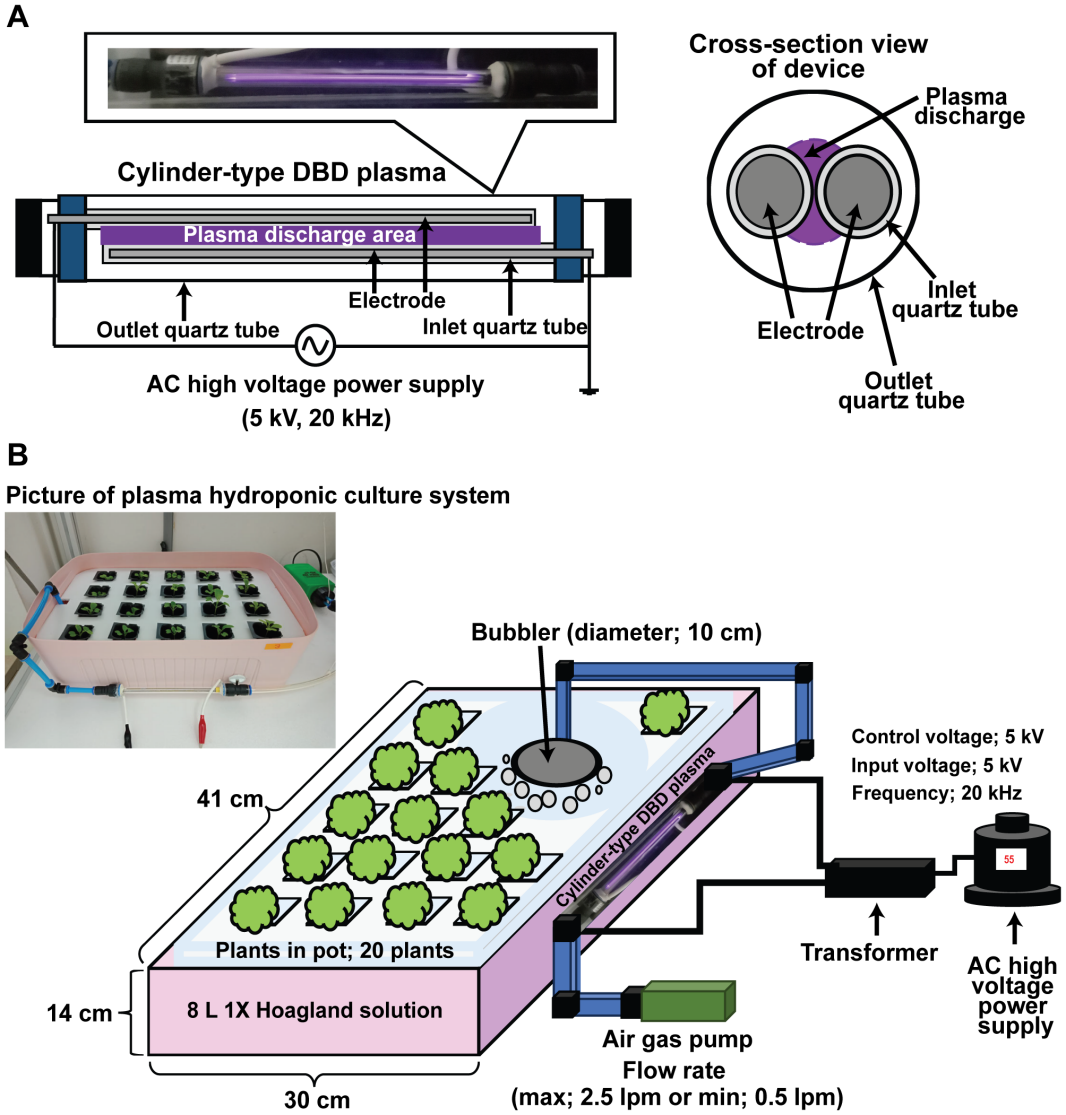
Hydroponic culture system with cylindrical electrode DBD plasma. Schematic views and photographs of the cylindrical electrode DBD plasma device **(A)** and hydroponic culture system **(B)**.

Bok choy seeds (Dong-Won Nong-San Seed Co., LTD., Yongin-si, Gyeonggi-do, Korea) were purchased and individually placed in sponge blocks (25 × 25 × 30 mm) soaked with deionized (DI) water for germination, incubated at 25°C in the dark for 1 week. After germination, the sponge blocks containing germinated seeds were transferred to a square pot each (39 × 39 × 45 mm). These were placed in Styrofoam plates (20 pots per plate) inside plastic containers (41 × 30 × 14 cm) containing 8 L of 1X Hoagland solution (**Figure 1A**). The Hoagland solution was prepared using DI water, as described in a previous study (Hoagland and Arnon, 1950). Bok choy plants were cultured with 1X Hoagland solution and aeration (**Figure 1B**).

In this study, we used the cylinder-type DBD plasma as the same plasma device used in a previous study (Ji et al., 2018), employing single-pair cylindrical DBD electrodes to generate plasma with air as the feeding gas (**Figure 1A**). Two brass electrodes (190 × 1 mm each) covered with quartz tubes (200 × 5 mm) were alternately connected to a high voltage or the ground with a control voltage of 55 kV, input voltage of 5 kV at a repetition frequency of about 20 kHz (**Figures 1A, B**). The electrode sets were placed in a larger cylindrical quartz sleeve (210 × 10 mm), with a distance of 1 mm between the electrodes (**Figure 1A**). Plasma was produced between them, using air fed with a fish air pump (Dae-Kwang Electronics, Inc., Seoul, Korea) at maximum flow (2.5 L/min), and the resulted gas was injected into the Hoagland solution through a bubbler for indicated times (**Figure 1B**).

One week old bok choy seedlings were cultured in Hoagland solution injected with plasma gas for 0 (control), 5 or 10 min, using bubbler at the maximum flow rate (2.5 L/min) and then with only air at the minimum flow rate (0.5 L/min) for 1 week. The solution was then discarded, after which 8 L of new Hoagland solution were placed in the container. New Hoagland solution was treated in the same way, with plasma gas for 0, 5 or 10 min and then with only air, and the plants were cultured for another week. Repeating this process, bok choy seedlings were cultured for a total of 4 weeks, replacing the Hoagland solution 3 times. Plants were cultured under a 16:8 h light: darkness cycle and 25°C.

### 2.2 Measurement of plant growth

The bok choy plants were harvested after 4 weeks and washed with DI water. After drying the plants with tissue paper, the shoot and root lengths were measured using a ruler, while the number of leaves per plant was counted. To obtain the dry weight (DW) of harvested individual plants, plants were placed in paper bags and dried using a dry oven at 65°C for 3–4 days. Subsequently, the dried plants were weighed using a balance (Kern, Albstadt, Ebingen, Germany).

### 2.3 Characterization of plasma gas-treated Hoagland solution

The physicochemical properties of the plasma gas-treated Hoagland solution were immediately characterized after treatment by measuring the concentration of H_2_O_2_, nitrogen oxide (NOx), hydroxyl radicals, ozone (O_3_), pH, electrical conductivity (EC), and oxidation reduction potential (ORP). For these analyses, Hoagland solution (8 L) was treated with plasma gas for 5 or 10 min, while the untreated solution (0 min) was used as a control. Following plasma treatment, the levels of H_2_O_2_ and NOx in the treated solutions were assayed using the Amplex™ Red Hydrogen Peroxide/Peroxidase Assay Kit (Molecular Probes, Eugene, OR, USA) and QuantiChromTM Nitric Oxide Assay Kit (BioAssay Systems, Hayward, CA, USA), respectively, following the manufacturer protocols.

To measure the hydroxyl radical level, terephthalic acid (Sigma-Aldrich, St. Louis, MO, USA) dissolved in 50 mM NaOH was incorporated into DI water or Hoagland solution until reaching a final concentration of 20 mM terephthalic acid. Subsequently, the DI water or Hoagland solution containing terephthalic acid was injected with plasma gas for 0, 5, or 10 min. Terephthalic acid can only react with hydroxyl radicals to produce 2- hydroxy terephthalic acid, which is fluorescent (Kanazawa et al., 2011); its fluorescence was detected at 310/425 nm (excitation/emission) using the Synergy HTX Multi-Mode Reader (BioTek Instruments, Winooski, VT, USA).

The O_3_ level, pH, EC, and ORP were measured using an ozone meter (CLEAN DOZ30 Dissolved Ozone Tester, Clean, Shanghai, China), pH meter (pH Testr^®^ 30 Pocket Testers; Oakton), EC meter (PCTS TestTM 50; Oakton), and ORP meter (ExStik™ Model RE300 Waterproof ORP Meter, Extech, Hudson, NH, USA), respectively. Levels of NO_2_ **^−^** and NO_3_ **^−^** ions in the plasma gas-treated Hoagland solution were analyzed using ion chromatography. To do so, the treated Hoagland solution was filtered (0.5-μm pore size) and injected into the ion chromatograph ICS-3000 (Thermo Scientific Dionex, Sunnyvale, CA, USA).

### 2.4 Measurement of chlorophyll and total soluble protein levels

Chlorophyll is a critical photosynthetic pigment that influences photosynthetic capacity and plant growth. To measure the chlorophyll levels, fresh leaves (0.2 g) of 4-week-old bok choy plants grown in plasma gas-treated Hoagland solution were cut into small pieces and placed into 50-mL conical tubes. For chlorophyll extraction, 20 mL of 80% acetone was added into each conical tube, after which the tubes were inverted multiple times to ensure that all leaves were properly mixed with the acetone solution. The tubes were covered with aluminum foil to prevent light exposure and incubated at 25°C until all leaves were completely white. Subsequently, the absorbance of the extracted liquid was measured at 663 and 645 nm using the Synergy HTX Multi-Mode Reader. The concentrations of chlorophyll a and chlorophyll b, together with the total chlorophyll in leaves of bok choy were calculated as mg/g fresh weight (FW) of leaves using the following equations (Manolopoulou et al., 2016):


$$Chlorophyll\;a\;\left(mg/g\;FW\right)=\left(12.7\times A663-2.69\times A645\right)\times \left(\frac{X}{1000}\right)\times n$$

$$\text{Chlorophyll b}\;\left(\text{mg}/\text{g FW}\right)=\left(22.9\times \text{A}645-4.68\times \text{A}663\right)\times \left(\frac{\text{X}}{1000}\right)\times \text{n}$$

$$\text{Total chlorophyll}\;(\text{mg}/\text{g FW})=\left(20.2\times \text{A}645-8.02\times \text{A}663\right)\times \left(\frac{\text{X}}{1000}\right)\times \text{n}$$

where A645 and A663 represent the absorption values at 645 and 663 nm, respectively, X is the total volume of liquid extract (mL), and n is the leaf FW (g).

The total soluble protein content was obtained from the shoots and roots of 4-week-old bok choy plants. The soluble protein content in plant cells is an indirect indicator of plant physiological and biochemical status, which contributes to plant growth. A 100-mg portion from each fresh shoot and root sample were ground in liquid nitrogen, after which the ground powder was transferred to 1.5-mL microtubes. Subsequently, 1 mL of 1X phosphate buffered saline (PBS) was added into the tube, and the tube was vortexed and then centrifuged at 12,400 × *g* and 4°C for 10 min (Song et al., 2015). The supernatants were collected and transferred into new tubes, where the Bradford assay kit (Bio-Rad, Hercules, CA, USA) was used to determine the concentration of total soluble protein following the manufacturer’s protocol. Bovine serum albumin was used as a standard.

### 2.5 Determination of NO_3_^−^-N and NH_4_^+^ content in the shoot and root

The primary nitrogen sources that plants can take up and utilize are NH_4_^+^ and NO_3_ **^−^** ions, so intracellular levels of NH_4_^+^ and NO_3_ **^−^**-N may be related to plant growth and quality. To determine the concentration of NO_3_ **^−^**-N and NH_4_^+^, shoots and roots were collected from plants cultured in the untreated and plasma gas-treated Hoagland solution. The samples were completely dried in the oven at 65°C and later ground in a mortar using a pestle. To determine the NO_3_ **^−^**-N level, 100 mg of shoot or root powder were suspended in 10 mL of DI water and incubated at 45°C for 1 h. After incubation, the suspensions were filtered (Whatman No. 40 filter paper, Whatman Inc., Maidstone, UK) and immediately analyzed for NO_3_ **^−^**-N levels using a salicylic acid-sulfuric acid method that provides the nitrosalicylic acid content (Lastra, 2003).

The phenol-hypochlorite reaction was used to analyze the level of NH_4_^+^ (King et al., 1990). The ground powder (10 mg) of shoot or root samples was mixed with 1 mL of DI water and shaken for 15 min at 25°C to extract the NH_4_^+^. This mixture was centrifuged at 12,400 × *g* for 5 min, after which the supernatant was transferred into new tubes. To determine the NH_4_^+^ concentration, a reaction mixture was constructed with 0.1 mL of supernatant, 0.4 mL of DI water, 2.5 ml of phenol-sodium nitroprusside (100 mM phenol and 0.16 mM sodium nitroprusside), and 2.5 ml of alkaline hypochlorite (125 mM NaOH and 5 ppm sodium hypochlorite solution) and incubated at 30°C for 10 min. After incubation, absorbance at 635 nm was measured using the Synergy HTX Multi-Mode Reader. Ammonium sulfate was used to make a standard curve from which the NH_4_^+^ level was inferred.

### 2.6 Response to salinity stress after plasma treatment

We analyzed the expression of genes associated with salinity stress tolerance. The mRNA expression levels of these genes were measured in bok choy plants from the treated and untreated groups. Shoots and roots of 4-week-old bok choy plants were harvested, washed with DI water, and stored at −80°C for further use. The shoots and roots were ground with liquid nitrogen, and the total RNA was extracted using the RNAiso Plus kit (Takara Bio, Shiga, Japan) according to the manufacturer’s instructions. The concentration of total RNA was measured using a NanoDrop spectrophotometer (BioTek Instruments), while 100 ng of total RNA were used to synthesize cDNA in the ReverTra Ace qPCR RT Master Mix with gDNA Remover (Toyobo, Osaka, Japan) according to the manufacturer’s instructions. The cDNA of three salinity stress tolerance-related genes—heptahelical protein, *HHP3*; WRKY transcription factor, *WRKY2*; and ABA-insensitive 1, *ABI1*—was further amplified to determine cycle threshold (Ct) values using an iQ SYBR Green Supermix (Bio-Rad) and CFX96TM real-time RT-PCR system (Bio-Rad). The thermal cycling conditions were as follows: 95°C for 3 min, 40 cycles at 95°C for 10 s and 60°C for 30 s. Using the Ct values, the relative levels of mRNA for the target genes compared to those of an actin gene (reference gene) were calculated as follows (Livak and Schmittgen, 2001):


$$Ratio=2^{-\Delta \Delta Ct},$$

$$-\Delta \Delta Ct={\left(Ct_{target}-Ct_{reference}\right)}_{control}-{\left(Ct_{target}-Ct_{reference}\right)}_{treatment}$$

The primer sequences used for qPCR are listed in **Table 1**. An average of three replicate measurements was obtained in each experiment, while the experiment was performed in triplicate.

**Table 1:** Primers used in a quantitative polymerase chain reaction (qPCR) for salinity stress-related gene expressions.

Primer name	Sequence (5’→ 3’)	Reference
HHP3-F	CAGAGACACCTTCCTTAGT	(Wang et al., 2019)
HHP3-R	TTACCACCATCATCCACAT	
WRKY2-F	CGGTTACTCGTTCGGTTTAGG	(Tang et al., 2013)
WRKY2-R	CGGTTGAGTCATATACGGGTG	
ABI1-F	AACTGCCCTTCCTTTGTCC	(Kong et al., 2018)
ABI1-R	AGGAATGATCGACGGTTTCA	
Actin-F	CTCAGTCCAAAAGAGGTATTCT	(Wang et al., 2019)
Actin-R	GTAGAATGTGTGATGCCAGATC	

The growth of bok choy plants under salinity stress in untreated or plasma gas-treated Hoagland solution was analyzed. Bok choy seeds were germinated for 1 week as described in section 2.1, after which they were transferred to plastic containers containing Hoagland solution (8 L) supplemented with 20 mM NaCl and incubated for 1 week. These solutions were either treated with air (control) or plasma gas for 10 min. After 1 week, the Hoagland solution was replaced with new Hoagland solution treated in the same way, and the bok choy plants continued to be cultured for 1 week. The same process was repeated once again; thus, the bok choy plants were cultivated for 4 weeks in total, renewing the Hoagland solution each week. All plants were grown under a 16:8 h light: darkness cycle at 25°C. After 4 weeks of culture, plant length and leaf number were measured.

### 2.7 Intracellular NO in bok choy root

To understand the potential mechanisms of plant growth enhancement in plasma gas-treated nutrient solutions, the intracellular NO levels in plant roots were examined. Intracellular NO is a signaling molecule involved in the regulation of plant growth, development, and immunity (Neill et al., 2003) (Domingos et al., 2015; Khan et al., 2023). Bok choy seeds were germinated and transferred into plastic containers filled with Hoagland solution, as described in section 2.1. Following plasma gas treatment of the Hoagland solution for 0 or 10 min, the bok choy plants were cultivated for an additional week. The plants were then harvested and washed with DI water to remove any organic matter adhered to the roots. Subsequently, the roots were soaked in 300 μL of 10 μM 4-amino-5-methylamino-2′7′-dichlorofluorescein diacetate (DAF-FM DA, Invitrogen, Waltham, MA, USA) in PBS (pH 7.5) and incubated in the dark for 1 h at 25°C. The samples were washed three times with fresh 1X PBS and mounted on glass slides. Observations (excitation, 495 nm; emission, 515 nm) were immediately conducted using an Olympus IX83-FP confocal microscope (Olympus, Tokyo, Japan).

### 2.8 Statistical analysis

All data are presented as the average of 9–20 replicates ± standard deviation. All experiments were repeated two or three times, with at least three replicate measurements performed for each experiment. The significance of differences observed in datasets was tested by a one-way ANOVA followed by *post hoc* Tukey’s HSD test at *p* < 0.001 (***), *p* < 0.01 (**), and *p* < 0.05 (*) using R software version 4.4.1 (The R foundation).

## 3 Results

### 3.1 Characterization of plasma gas-treated Hoagland solution

**Figure 2:**
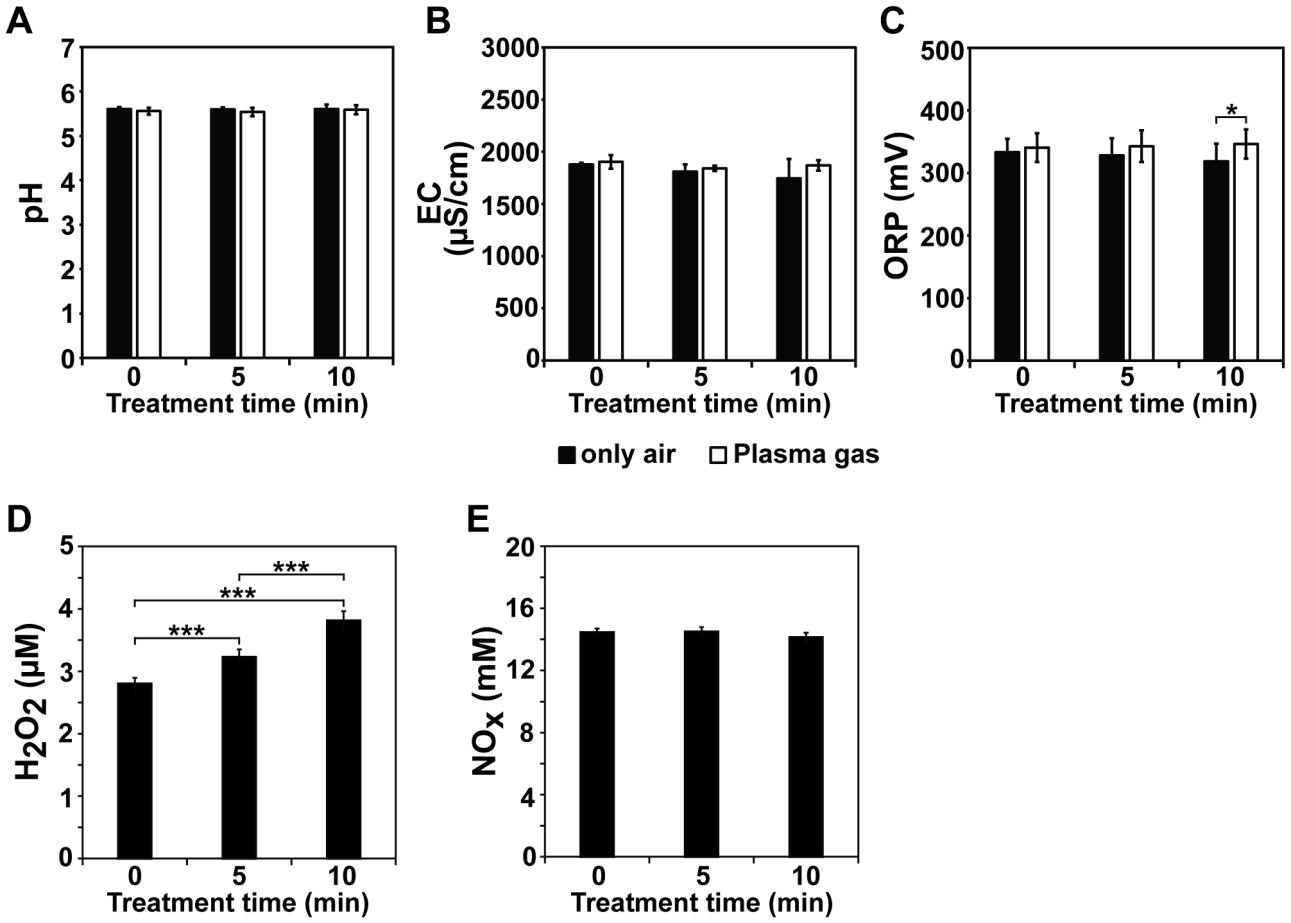
Analysis of physicochemical properties of Hoagland solution after plasma gas treatment. Average pH **(A)**, EC **(B)**, ORP **(C)**, H_2_O_2_ **(D)** and NOx **(E)** levels measured in Hoagland solution injected with plasma gas for 0, 5, and 10 min. Each value represents the mean and standard deviations of replicate measurements; n = 6 or n = 9. ***p < 0.001 and *p < 0.05.

The electrical current and voltage of the discharged plasma and its active species analyzed using optical emission spectroscopy were described in a previous study (Ji et al., 2018). The physicochemical properties of the plasma gas-treated Hoagland solution, including the pH, ORP, EC, and H_2_O_2_, NOx, hydroxyl radical, and O_3_ levels, were measured. The pH of the Hoagland solution was not dramatically changed after plasma gas treatment, with 5.60–5.61 and 5.53–5.59 pH values measured in the non-treated (only air) and plasma gas-treated Hoagland solutions, respectively (**Figure 2A**). The EC values were slightly increased after plasma gas treatment of Hoagland, but there were no significant differences with only air-gas-treated Hoagland (**Figure 2B**). In the air-treated and plasma gas-treated solutions, EC values of approximately 1809 and 1843 µS/cm (5 min) and 1745 and 1869 µS/cm (10 min) were detected, respectively (**Figure 2B**). Similarly, the ORP slightly increased after plasma gas treatment (**Figure 2C**). It was significantly higher (p < 0.05) in Hoagland treated with plasma gas (346 mV) than that treated with air (319 mV) for 10 min (**Figure 2C**) while no significant differences between only air- and plasma gas-treated Hoagland for 5 min, values of approximately 328 and 343 mV, respectively (**Figure 2C**).

Owing to limitations in the available methodologies, we measured the concentrations of H_2_O_2_, NOx (including NO, NO_2_ **^−^**, and NO_3_ **^−^**, as NO is rapidly oxidized to NO_2_ **^−^** and NO_3_ **^−^**), hydroxyl radicals, and O_3_ in Hoagland solution after plasma gas treatment. The H_2_O_2_ level in the Hoagland solution increased after plasma gas treatment in a time-dependent manner (**Figure 2D**). Under plasma gas-treatment for 5 and 10 min, the H_2_O_2_ concentrations in the solution were approximately 3.23 and 3.81 µM, respectively, and significantly higher than that in the untreated control (0 min), at 2.80 µM (*p* < 0.001) (**Figure 2D**). In addition, the H_2_O_2_ level in the Hoagland solution increased significantly (p < 0.001) after 10 min of plasma gas treatment compared to 5 min of plasma treatment (**Figure 2D**). The level of NOx (including NO, NO_2_ **^−^**, and NO_3_ **^−^**) was not significantly different between the plasma gas-treated and untreated Hoagland solutions (**Figure 2E**). Approximately 14.46 mM, 14.49 mM, and 14.13 mM NOx were observed in Hoagland solution treated for 0, 5, and 10 min, respectively (**Figure 2E**). Ion chromatography showed that the NO_3_ **^−^** level was slightly increased after plasma gas treatment, from 739.4 (0 min) to 788–795.4 mg/L (5 and 10 min), whereas NO_2_ **^−^** was not detected (**Table 2**; **Supplementary Figure S1**).

**Table 2:** Level of anions in Hoagland solution after plasma gas treatment according to ion chromatography.

Treatment time	Negative ions (mg/L)		
	NO_3_^−^	SO_4_^2 −^	PO_4_^3 −^
0 min	739.4^*^	209.6	73.5
5 min	788.0	222.8	78.9
10 min	795.4	224.3	77.9

*Each value was assessed from one experimental measurement.

Neither hydroxyl radicals nor O_3_ were detected in the treated and untreated Hoagland solutions. However, 0.28 µM and 0.61 µM values of hydroxyl radicals were measured in the DI water treated with plasma gas for 5 and 10 min, respectively (**Supplementary Figure S2**).

### 3.2 Bok choy plant growth was enhanced in plasma gas-treated Hoagland

**Figure 3:**
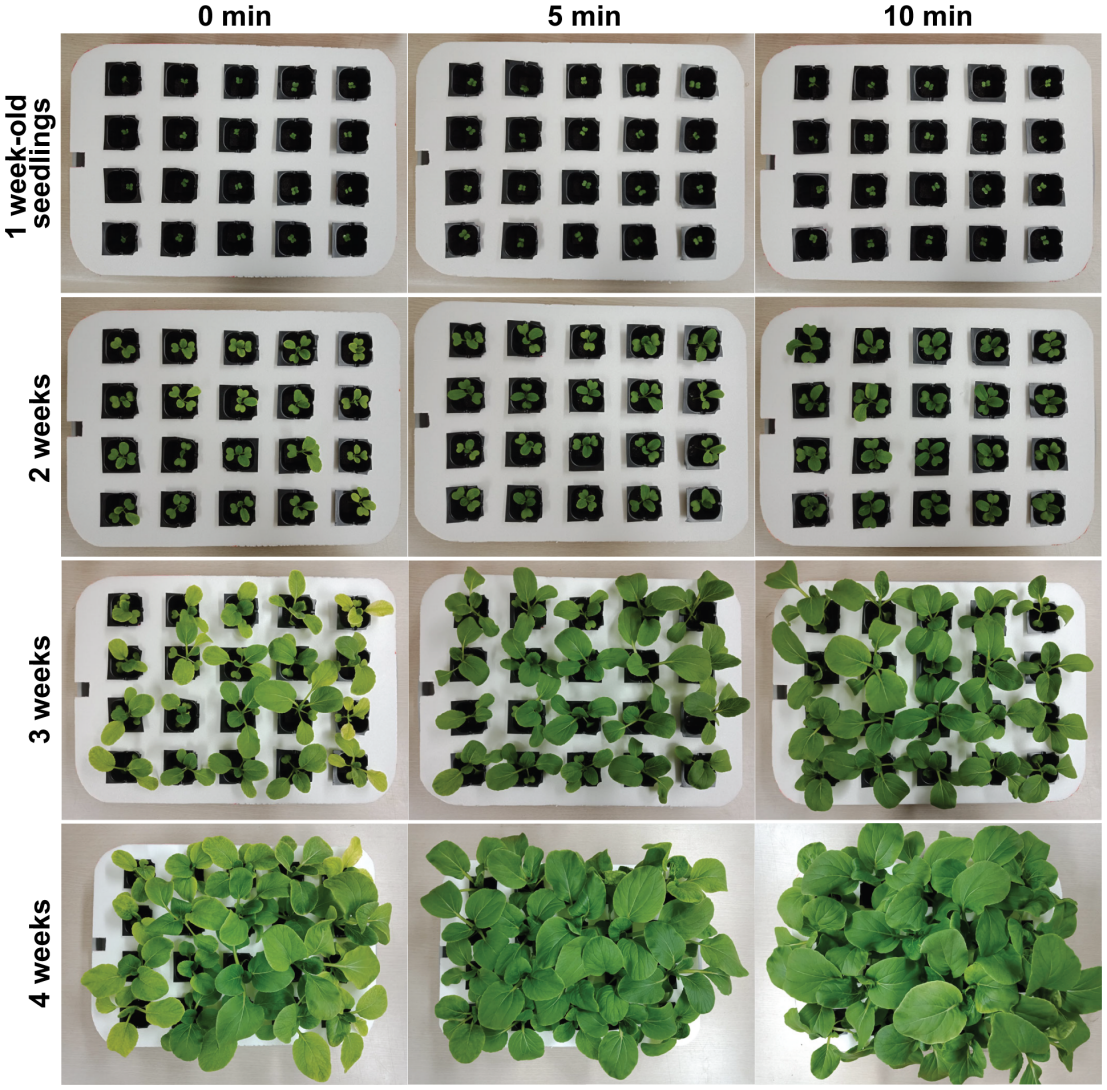
Photograph of bok choy plants grown in Hoagland solution injected with plasma gas for 0, 5, and 10 min in the hydroponic culture system.

**Figure 4:**
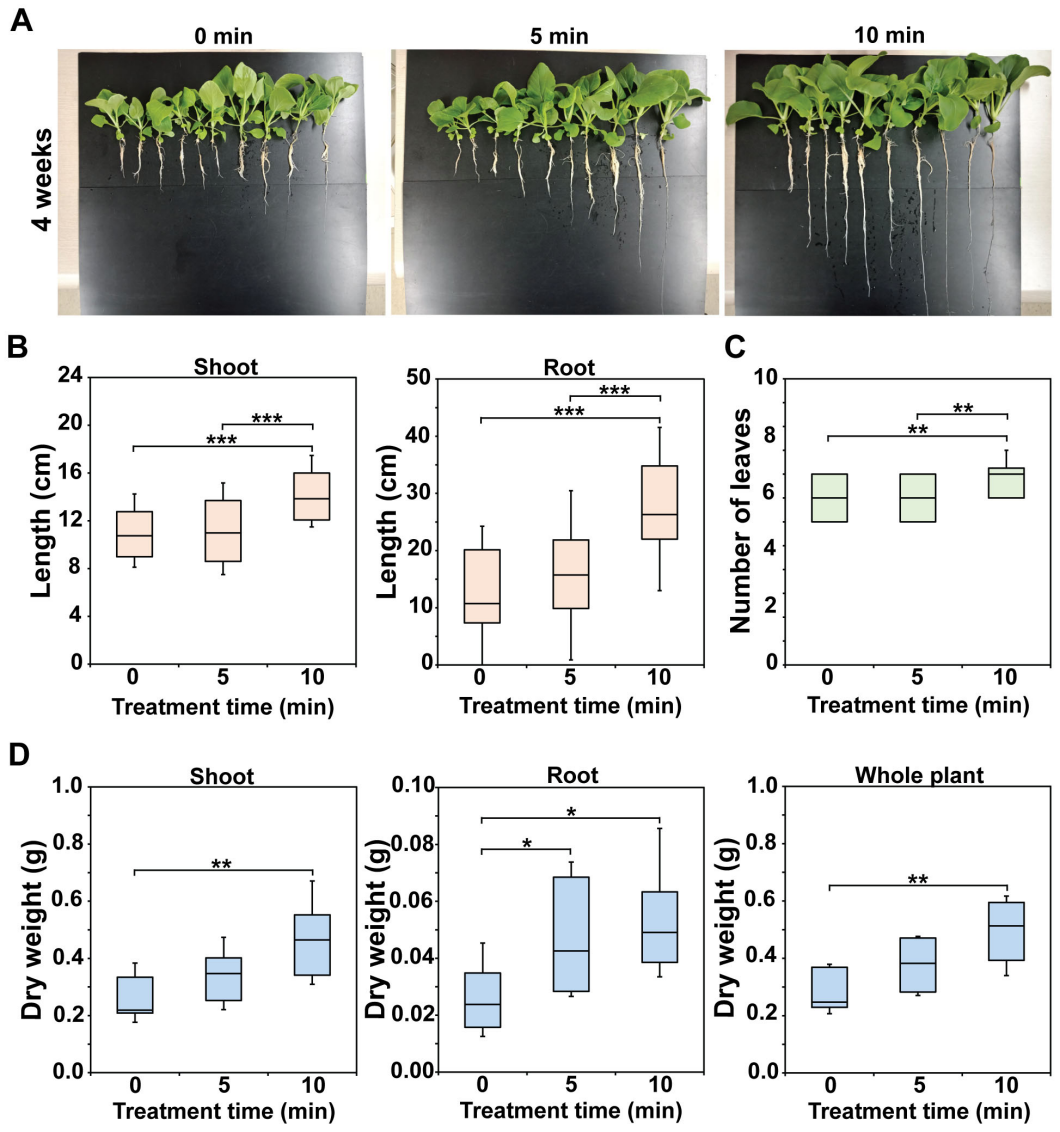
Morphometric parameters of 4-week-old bok choy plants. Photograph of harvested bok choy plants grown for 4 weeks in Hoagland solution injected with plasma gas for 0, 5, and 10 min **(A)**. Average length of shoots and roots **(B)**, number of leaves **(C)**, and dry weight **(D)** of individual plants. Each value represents the mean and standard deviations of replicate measurements; n = 36 plants **(B, C)** and n = 10 plants **(D)**. ***p < 0.001, **p < 0.01 and *p < 0.05.

**Figure 3** displays a photograph of bok choy plants in hydroponic pots after 1–4 weeks of cultivation, clearly showing that plants grew better in Hoagland treated with plasma gas—particularly that treated for 10 min—than in untreated Hoagland (0 min) (additional replicate data are shown in **Supplementary Figure S3**). The plants were harvested after 4 weeks (**Figure 4A**, additional replicate data in **Supplementary Figure S4**), when growth was quantitatively analyzed. Shoots and roots of bok choy grown in treated Hoagland were longer than those grown in untreated Hoagland (**Figures 4A, B**). Significantly longer shoot and root lengths (*p* < 0.001) were observed in the 10 min plasma gas-treated Hoagland plants compared to those of the untreated solution (**Figure 4B**), with increases of approximately 25.6% in shoot length (11.2 to 14.1 cm) and 97.2% in root length (13.8 to 27.2 cm) (**Figure 4B**). The number of leaves per plant was also significantly increased (*p* < 0.01) after the 10 min treatment (**Figure 4C**). Moreover, the average total dry weight (DW) of individual plants was significantly increased (*p* < 0.01) in Hoagland treated for 5 (36.8%) and 10 min (80.5%) compared to that in the untreated solution (**Figure 4D**). The DW of both shoots and roots was also higher for plants grown in plasma gas-treated Hoagland (**Figure 4D**). In particular, the DW of shoots grown in 10 min plasma gas-treated Hoagland was significantly greater (*p* < 0.01) than that of plants grown in the untreated solution (**Figure 4D**).

### 3.3 Chlorophyll and total soluble protein content and nitrogen uptake were enhanced in bok choy grown in plasma gas-treated Hoagland

**Figure 5:**
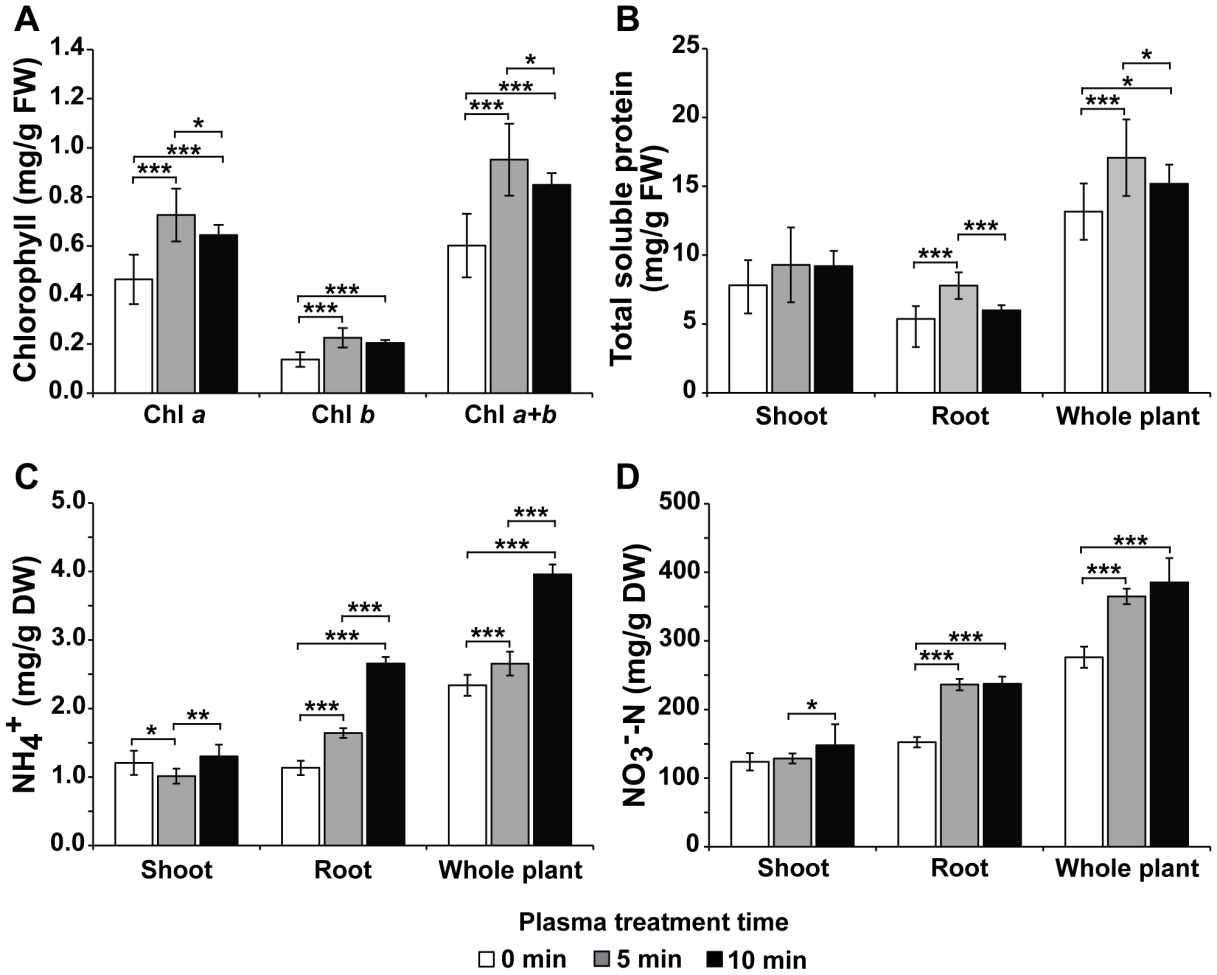
Average chlorophyll content **(A)**, total soluble protein concentration **(B)**, and NH_4_^+^ **(C)** and NO_3_ **^−^**-N **(D)** levels in 4-week-old bok choy plants grown in Hoagland solution injected with plasma gas for 0, 5, and 10 min. Each value represents the mean and standard deviations of replicate measurements; n =18 **(A, B)** and n = 9 **(C, D)**. ***p < 0.001, **p < 0.01 and *p < 0.05.

As plant growth was enhanced when Hoagland was treated with plasma gas, the physiological and biochemical statuses related to plant growth in cells were also investigated. The contents of chlorophyll a, chlorophyll b, and total chlorophyll per g of leaf fresh weight (FW) were significantly increased (*p* < 0.001) in the leaves of bok choy cultured in plasma gas-treated Hoagland solution for 4 weeks compared to those in plants cultured in untreated (0 min) Hoagland (**Figure 5A**). The total chlorophyll values in bok choy cultivated in Hoagland injected with plasma gas for 0, 5, and 10 min were 0.60, 0.95, and 0.85 mg/g FW, respectively (**Figure 5A**). Increases of approximately 58.2% (5 min) and 41.1% (10 min) were observed in total chlorophyll content of plants grown in treated Hoagland, compared to that of plants in untreated Hoagland (**Figure 5A**).

The total soluble protein content in the shoot, root, and entire plant was measured after cultivation in Hoagland solution for 4 weeks (**Figure 5B**). Generally, the total soluble protein content was higher in the shoots than in the roots (**Figure 5B**), while the content in roots was higher in plants grown in treated Hoagland (5 min) than that in plants grown in untreated (0 min) solution. Additionally, the total soluble protein content in the entire plant was significantly greater (*p* < 0.001 or *p* < 0.05) when plants were cultivated in plasma gas-treated Hoagland (5 min, 17.1 mg/g; 10 min, 15.2 mg/g FW) than in untreated solution (13.2 mg/g FW) (**Figure 5B**).

In general, both NH_4_^+^ and NO_3_ **^−^**-N concentrations were higher in the roots than in the shoots (**Figures 5C, D**). The levels of NH_4_^+^ were significantly increased (*p* < 0.001) in the roots of plants grown in Hoagland treated with plasma gas for 5 and 10 min (approximately 1.64 and 2.66 mg/g DW, respectively), compared to that of plants grown in untreated (0 min) solution (1.13 mg/g DW). Conversely, concentrations in the shoots were not significantly increase between the untreated and treated groups (**Figure 5C**). The NH_4_^+^ levels in the entire plant were considerably higher under cultivation with treated Hoagland (approximately 3.96 mg/g DW in 10 min treatment) than those measured in the untreated group (2.34 mg/g DW) (**Figure 5C**).

Regarding NO_3_ **^−^**-N accumulation, approximately 152.40, 236.27, and 237.34 mg/g DW were present in the roots of plants grown in Hoagland treated with plasma gas for 0 (untreated), 5, and 10 min, respectively (**Figure 5D**). Significantly greater (*p* < 0.001) NO_3_ **^−^**-N levels were observed in roots of plants grown in plasma gas-treated Hoagland compared with those grown in untreated Hoagland, whereas no significant differences were observed in NO_3_ **^−^**-N levels in the shoots (**Figure 5D**). Furthermore, concentrations of NO_3_ **^−^**-N in the entire plant were higher when Hoagland was treated with plasma gas than when there was no treatment, with the highest accumulation observed in the group treated for 10 min (approximately 385.20 mg/g DW) (**Figure 5D**).

### 3.4 Cultivation in plasma gas-treated Hoagland improved plant tolerance to salinity stress

**Figure 6:**
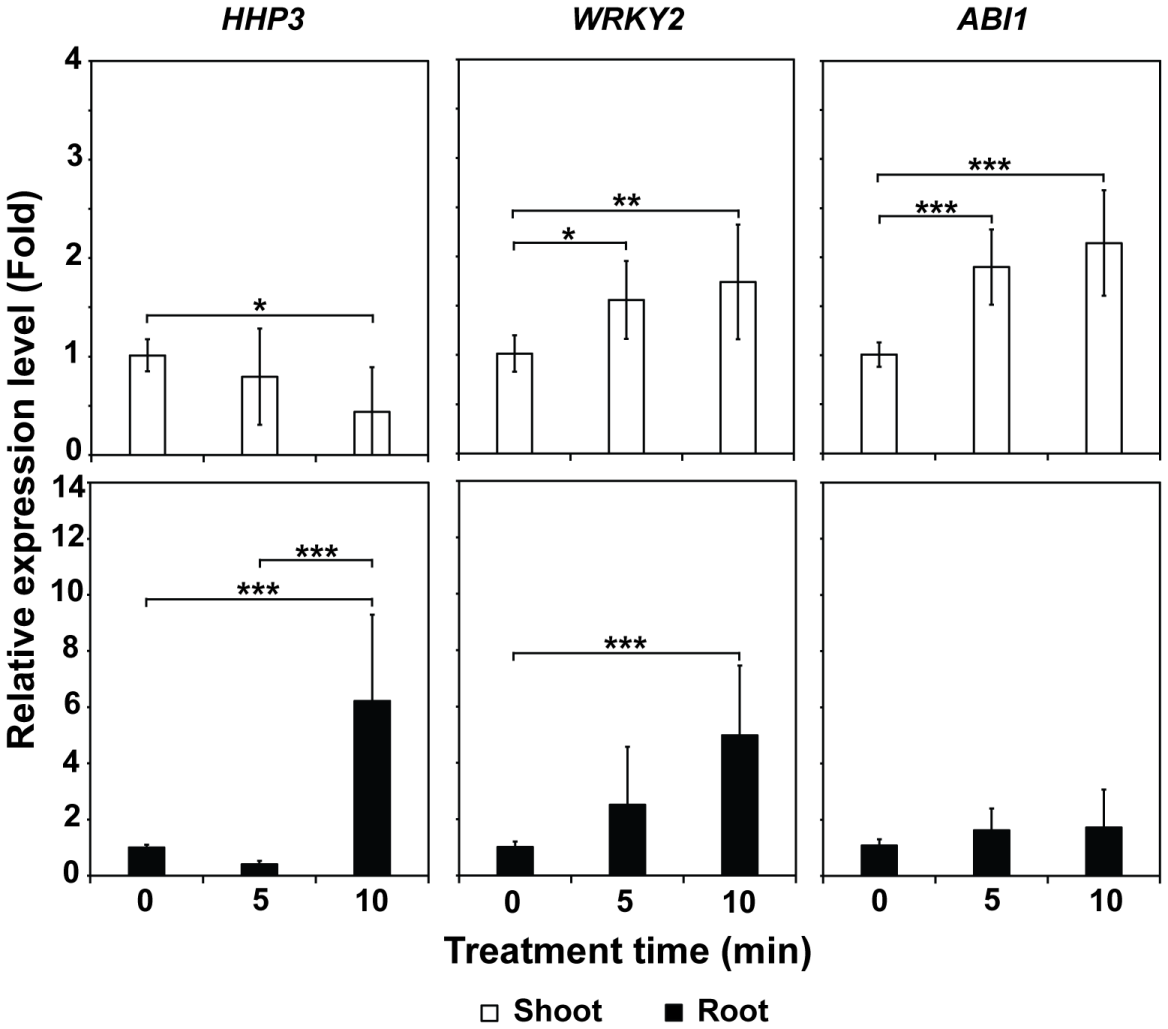
Average mRNA levels of three salinity stress-related genes—*HHP3*, *WRKY2*, and *ABI1*—in shoots and roots of 4-week-old bok choy plants grown in Hoagland solution injected with plasma for 0, 5, and 10 min. Each value represents the mean and standard deviations of replicate measurements; n = 6 or n = 9. ***p < 0.001, **p < 0.01 and *p < 0.05.

The mRNA levels of the *WRKY2* and *ABI1* genes were significantly elevated (*p* < 0.001, *p* < 0.01, and *p* < 0.05) in the shoots of plants grown in treated Hoagland compared to those in plants grown in untreated (0 min) solution (**Figure 6**). In the roots, the mRNA levels of *HHP3* and *WRKY2* were significantly higher (*p* < 0.001) in plants cultured in Hoagland injected with plasma gas for 10 min (approximately 6.2 and 5.0-fold, respectively) than for 0 min (untreated control) (**Figure 6**).

**Figure 7:**
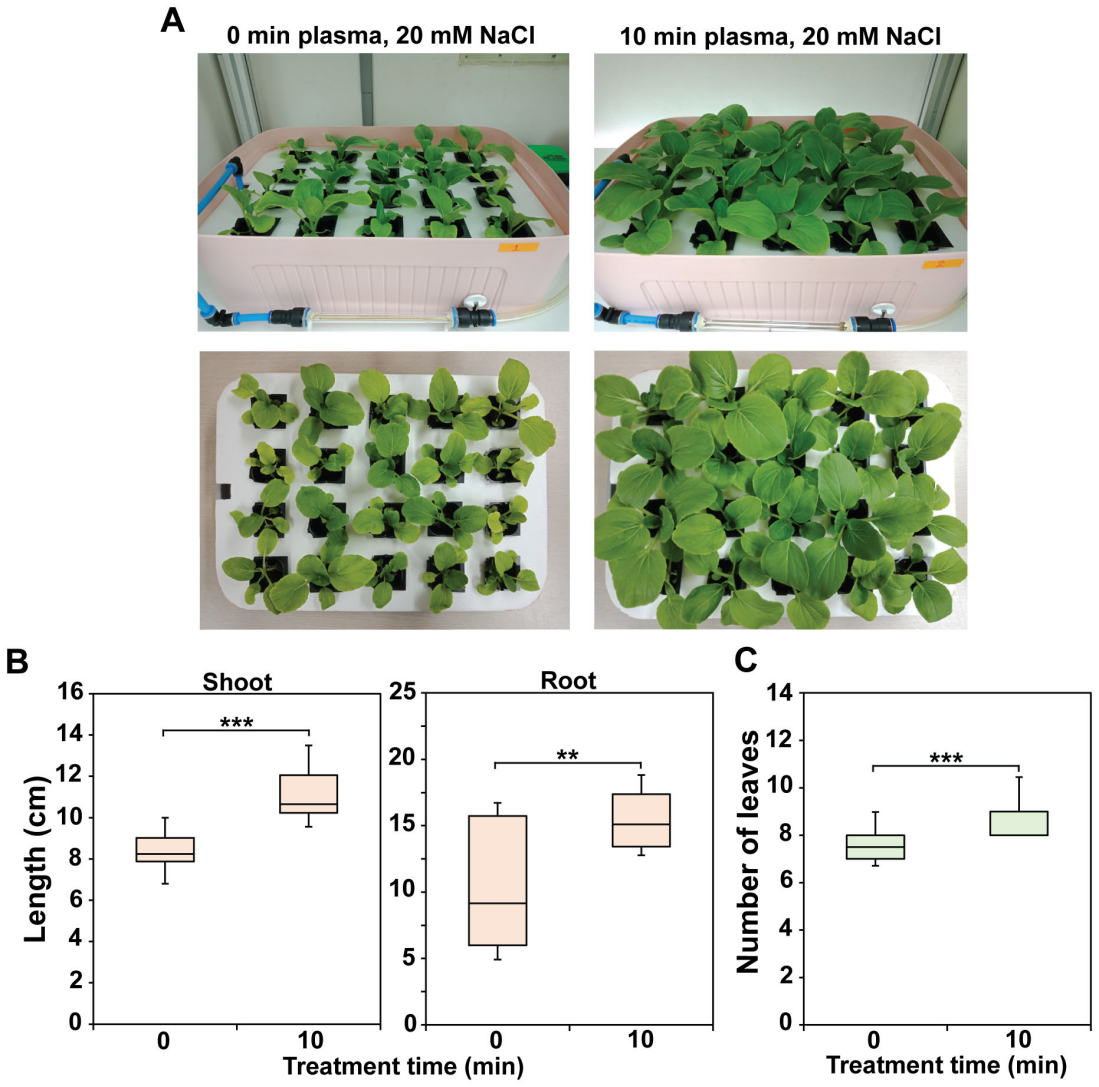
Morphometric parameters of 4-week-old bok choy plants cultured in untreated (0 min) or plasma gas-treated (10 min) Hoagland under salinity stress (20 mM NaCl). **(A)** Photograph of bok choy plants cultured in Hoagland solution. Average shoot and root lengths **(B)** and number of leaves **(C)** of individual plants grown for 4 weeks. Each value represents the mean and standard deviations of replicate measurements; n = 20. ***p < 0.001 and **p < 0.01.

**Figure 7A** shows a photograph of 4-week-old bok choy plants cultivated in Hoagland solution containing 20 mM NaCl, injected with plasma gas for 0 or 10 min once a week (additional replicate data are shown in **Supplementary Figure S5**). Direct observation demonstrated that plants grew faster in plasma gas-treated Hoagland than in untreated solution under salinity stress (**Figure 7A**). The shoots and roots were significantly longer (*p* < 0.001 or *p* < 0.01) in individual plants grown in treated Hoagland than in the untreated solution (**Figure 7B**). Additionally, the number of leaves per plant was significantly greater (*p* < 0.001) in individual plants grown in the treated solution than in those grown in untreated Hoagland (**Figure 7C**).

### 3.5 Intracellular NO level in bok choy roots

**Figure 8:**
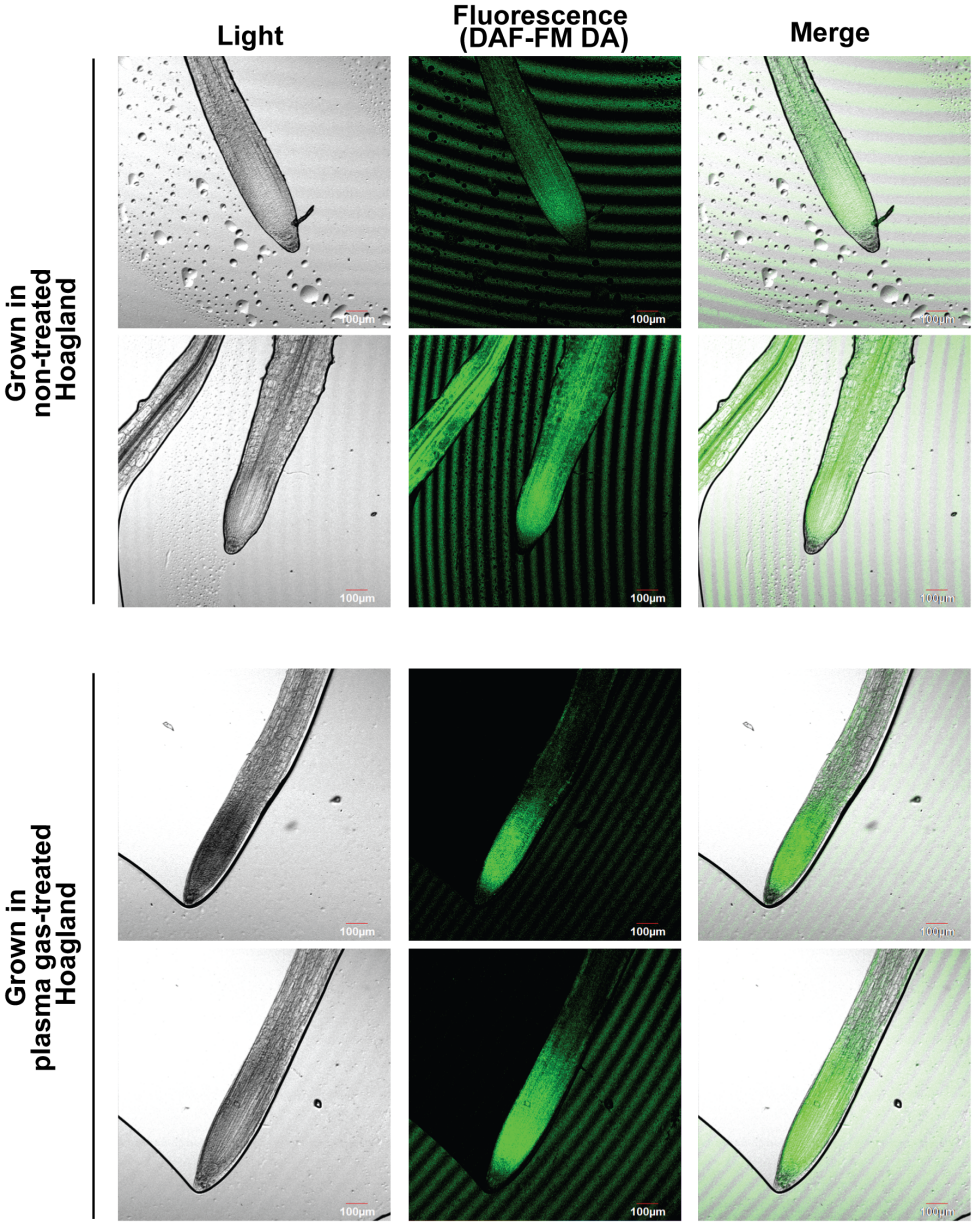
Results of assay for intracellular NO in roots of bok choy plants grown in untreated (0 min) or plasma gas-treated (10 min) Hoagland solution.

Fluorescence (indication of intracellular NO) was observed in a larger area of the roots of plants grown in untreated Hoagland than in those grown in plasma gas-treated Hoagland (**Figure 8**; **Supplementary Figure S6**). In the roots of plant grown in Hoagland injected with plasma gas for 10 min, fluorescence was strongly detected close to the zone of cell division and elongation (**Figure 8**; **Supplementary Figure S6**).

## 4 Discussion

The results of this study suggest that treating nutrient solutions with plasma gas may improve plant growth as well as the physiological and biochemical processes in hydroponic cultures. Improvements in seed germination and plant growth following treatment with plasma or plasma treated water have often been reported in soil culture systems (Lo Porto et al., 2018; Rasooli et al., 2021; Rathore et al., 2022; Yemeli et al., 2022; Bian et al., 2024). Similarly, the enhancement of plant growth using plasma-treated water or nutrient solutions has also been reported for hydroponic culture systems in several studies. Baby-leaf lettuce (*Lactuca sativa* var. *acephala*) grown in a hydroponic system using non-thermal plasma-treated nutrient solutions showed an increase in fresh leaf biomass, carotenoid, chlorophyll, and total phenol contents, and antioxidant capacity (Carmassi et al., 2022). A study on sweet basil (*Ocimum basilicum* L.) cultured in a hydroponic system with plasma-activated nutrient solution further resulted in increased plant height, greater fresh and dry mass, and higher greenness value than those of control plants (Date et al., 2023). Moreover, PAW has been described as a sustainable agricultural technology that improves seed germination, plant development, and biotic and abiotic stress tolerance (Gao et al., 2022).

Our results additionally showed that intracellular physiological and biochemical factors, such as chlorophyll content (photosynthetic activity), total soluble protein concentration (biochemical activity), and NH_4_^+^ and NO_3_^−^-N levels (nitrogen assimilation) were improved in plants grown in Hoagland treated with plasma gas. Nitrogen represents one of the most essential mineral nutrients for plant growth and biomass production and is a constituent element of nucleic acids, amino acids, proteins, lipids, chlorophyll, and numerous primary and secondary metabolites (Wang et al., 2014). In plants, nitrogen is absorbed by the roots in the form of NO_3_^−^ and NH_4_^+^, which are subsequently distributed throughout the entire plant to support its growth and development (Poothong and Reed, 2016; Cui et al., 2017; Tho et al., 2017). Nitrogen presence in plants is evidenced by darker green leaves, increased protein levels, and enhanced seed plumpness, ultimately boosting crop productivity (Fathi, 2022). In this study, bok choy plants grown in plasma gas-treated Hoagland solution has accumulated more NO_3_^−^ and NH_4_^+^ in the roots than plants in the control group, leading to increases in chlorophyll and soluble protein contents as well as in plant biomass. A previous investigation had also demonstrated that green oak lettuce (*Lactuca sativa* L.) cultivated with Hoagland solution in a hydroponics system exhibited greater accumulation of NO_3_^−^ in both shoots and roots when using a nitrate source produced by the pinhole plasma jet, leading to increased plant growth, yield, and amino acid accumulation (Ruamrungsri et al., 2023). Notably, however, the NOx concentration in the Hoagland solution did not change significantly after treatment with plasma gas in our study although nitrogen uptake into plant roots increased. Therefore, the higher intracellular level of NO_3_^−^ and NH_4_^+^ in plants grown in plasma gas-treated Hoagland may not have resulted from the NOx level in the nutrient solution. We suggest that the influx rate through NO_3_^−^ transporter in plant cells, potentially activated in plasma gas-treated Hoagland, may be elevated, resulting in similar levels of NO_3_^−^ in control and treated-nutrient solutions. However, we did not quantify the expression and activation level of NO_3_^−^ transporter in plant cells in this study, and further analysis might be required to examine this hypothesis. Our data also showed that there was a slight decrease or no significant change in chlorophyll contents in leaves of plants grown in between 5 min and 10 min plasma gas treated Hoagland solution. This indicates that 5 min longer treatment with plasma gas may not be able to cause any significant change in chlorophyll contents in leaves. No significant difference in plant biomass (dry weight) between 5 min and 10 min plasma gas treatments (as shown in **Figure 4D**) may have been resulted from no significant change in chlorophyll contents (level of photosynthesis). Although length of shoot and root and leaves number were significantly greater in 10 min than 5 min plasma gas treatment (as shown in **Figures 4B, C**), plant dry weight (biomass) could be more closely associated with photosynthetic capability. Therefore, no significant change in chlorophyll contents may have resulted in no significant change in plant biomass between 5 min and 10 min plasma gas treatments.

Several analyses were conducted in this study to determine the mechanism underlying the enhanced plant growth promoted by plasma gas-treated Hoagland solution. Non-thermal plasma generates reactive oxygen and nitrogen species, energetic electrons, and radiation in the gaseous phase, which are transferred to the liquid solution when the plasma interacts with it (Kaushik et al., 2019; Zhou et al., 2020; Shaji et al., 2023; Wong et al., 2023). We first measured the levels of H_2_O_2_, NOx, O_3_, and OH radicals in the Hoagland solution, finding that the H_2_O_2_ concentration was slightly found in the untreated Hoagland solution and elevated after plasma gas injection. Numerous studies have indicated that H_2_O_2_ can be produced at the water surface in aquatic environments due to photochemical reactions involving chromophoric dissolved organic matter (Cooper et al., 1988, 1994; O’Sullivan et al., 2005; Garcia et al., 2019). Likewise, the low amount of H_2_O_2_ detected in the untreated Hoagland solution could be attributed to the interaction between the components in the solution and light. Furthermore, there have been findings suggesting that the presence of Ni^2+^, F^−^, PO_4_^3−^, and CO_3_^2−^ can lead to a significant increase in the production of H_2_O_2_ (Wang et al., 2021). In our case, PO_4_^3−^ present in the untreated Hoagland solution may have contributed to generation of H_2_O_2_. Many studies have demonstrated that H_2_O_2_ is the long-lived reactive oxygen species discovered in liquid solutions following plasma treatment (Judée et al., 2018; Veerana et al., 2019; Xu et al., 2020). In plasma-treated solutions, H_2_O_2_ is mainly produced in two ways: (1) The combination of OH radicals created in the gas phase produces H_2_O_2_, which is then disseminated directly into the liquid solution, or (2) the OH radicals produced in the gas phase are released into the liquid, where they react with liquid molecules to form H_2_O_2_ (Kim et al., 2014; Kovacevic et al., 2017). In this study, OH radicals were detected in neither the untreated nor plasma gas-treated Hoagland solutions. Considering that an increase in OH radicals was observed in plasma gas-treated DI water (**Supplementary Figure S2**), we suggest that OH radicals may have been generated in the plasma gas-treated Hoagland solution but quickly consumed to produce H_2_O_2_. Alternatively, OH radicals may be scavenged in the Hoagland solution, likely through a reaction with its components or the components in the Hoagland solution may interfere with the terephthalic acid reaction, which is used to detect OH radicals.

The increase in H_2_O_2_ levels in Hoagland solution treated with plasma gas is likely to have contributed to the promotion of growth and the physiological and biochemical processes of bok choy plants in our study. Currently, H_2_O_2_ is considered to have a significant impact on plant growth and physiological processes such as seed germination, root system development, flowering, stomatal aperture regulation, senescence, and programmed cell death (Niu and Liao, 2016). A previous study reported that spraying *Ficus deltoidei* plants with 16 and 30 mM H_2_O_2_ once a week results in significant increases in plant height, leaf area, net photosynthetic rate, stomatal conductance, chlorophyll content, and quantum yield (Nurnaeimah et al., 2020). In cucumber, spraying leaves with 1.5 mM H_2_O_2_ significantly increased the leaf relative water content, biomass, chlorophyll content, and net photosynthetic rate (Sun et al., 2016). In addition, maize (*Zea mays* L.) cultivars grown in Hoagland solution containing 0.5 mM H_2_O_2_ showed increased growth and water, proline, mineral, total soluble protein, and total sugar contents in leaves compared to those of a control (Guzel and Terzi, 2013). Exogenous H_2_O_2_ at low concentrations acts as a signaling molecule that promotes various physiological processes, including seed germination, stomatal opening, chlorophyll content, and senescence delays; however, at high concentrations, it can cause oxidative damage to biomolecules, which may result in cell death (Cerny et al., 2018; Nazir et al., 2020). Therefore, our results suggest that the H_2_O_2_ generated in the Hoagland solution after treatment with plasma gas is a suitable level that could function as a signaling molecule that promotes growth and physiological processes in bok choy.

Physicochemical properties measured in the Hoagland solution, such as pH, EC, and ORP, did not exhibit changes after plasma gas treatment. The lack of significant changes in pH values may be attributed to the buffering activity and/or metal ions in the Hoagland solution maintaining the pH in the neutral range. Recent studies have shown that adding metal ions such as magnesium (Mg^2+^), zinc (Zn^2+^), and aluminum (Al^3+^) to water before plasma treatment can improve its pH compared to that resulting from plasma treatment without metal ions (Lamichhane et al., 2021; Javed et al., 2023). As the Hoagland solution contains numerous metal ion components, these may have contributed to pH regulation in the solution.

Interestingly, we observed that intracellular NO in bok choy roots grown in plasma gas-treated Hoagland solution was strongly detected in the zone of cell division and elongation, which is related to root growth; however, in the control group, it was dispersed in a broad area of the roots. This could be related to the increased NO_3_^−^ uptake in roots of bok choy grown in plasma gas-treated Hoagland solution, as intracellular NO can be synthesized through reduction of NO_3_^−^ and NO_2_^−^ during nitrate assimilation in plants (Wilson et al., 2008). Intracellular NO acts as a signaling molecule that regulates growth and development, stress tolerance, and immunity in plants (Neill et al., 2003; Domingos et al., 2015; Khan et al., 2023). Bok choy plants grown in plasma gas-treated Hoagland solution could potentially uptake more NO_3_^−^ than plants in the control group, generating intracellular NO in the cell division and elongation zone of the root via reduction of NO_2_^−^ during NO_3_^−^ assimilation.

Plants encounter various stresses in their natural environment; in particular, salinity is a major abiotic stressor that has detrimental effects on plant growth and development (Zhao et al., 2021). Our study suggests that using plasma gas-treated Hoagland solution in hydroponic cultures can promote the tolerance of plants to salinity stress. One experimental piece of evidence for tolerance enhancement in our study is the upregulation of several salinity stress-related genes—*WRKY2*, *ABI1*, and *HHP3*—in the shoots and roots of bok choy plants grown in plasma gas-treated Hoagland solution. The increased expression of *WRKY2*, *HHP3*, and *ABI1* has often been observed in bok choy in response to high salinity environments (Tang et al., 2013; Kong et al., 2018; Wang et al., 2019). WRKY is a well-known stress-related transcription factor that plays a crucial role in regulating various abiotic stresses encountered by many plants (Chen et al., 2012). Another experimental evidence is that bok choy growth in the presence of 20 mM NaCl was greater when plasma gas-treated Hoagland solution was used than when untreated Hoagland solution was the medium. Numerous studies have revealed that plasma-activated water or solutions can enable plants to withstand and adapt to various stressors (Gao et al., 2022). When it comes to salinity stress, PAW pretreatment can improve the salinity tolerance of barley, with H_2_O_2_ and NO playing crucial roles in this enhancement (Gierczik et al., 2020). In our study, we did not pre-treat plants with plasma before exposure to high salinity but placed plants in plasma treated solution under high salinity (treatment with plasma and high salinity at the same time). In addition, we did not monitor the change in level of NaCl (whether high salinity is maintained or not) in Hoagland solution during plant cultivation. Further investigations are still needed for better understanding the role of plasma in regulation of salt stress. Recent studies have shown that H_2_O_2_ plays a pivotal role as a signaling molecule in the pathway associated with abiotic stress responses (Silva et al., 2020). Moreover, H_2_O_2_ acts as a metabolic signal, facilitating the maintenance of ionic and redox homeostasis and enhancing plant tolerance to salinity-induced stress (Silva et al., 2020). Seed priming with H_2_O_2_ has enhanced the tolerance to salinity stress by boosting both enzymatic and non-enzymatic antioxidant defense mechanisms (Niu and Liao, 2016; Momeni et al., 2023). In our study the level of H_2_O_2_ was elevated in the plasma gas-treated Hoagland solution, which might play a role in enhancing the salinity tolerance of bok choy plants; further research is required to elucidate this issue.

Although positive effects of plasma gas treatment on hydroponic plant cultivation have been demonstrated, our study is still limited to the examination in a controlled laboratory environment using a plant species, bok choy, during seedling stage. The findings from our research provide valuable insights into the potential benefits of non-thermal plasma technology, but they are not sufficient to draw broad conclusions applicable to all hydroponic systems. To extend the use of non-thermal plasma technology to industrial-scale hydroponic cultivation, it is essential to conduct additional research on various plant species under diverse environmental conditions. In addition, the sustainability of utilizing non-thermal plasma in large-scale cultivation and the implications of plasma gas treatment for future commercial use require comprehensive assessment. About the mechanisms of plasma effects, H_2_O_2_ might be one of critical factors for plasma mediated enhancement of plant growth in our study. However, other species or mechanisms are still possible for explanation of plasma effects, and further investigation is needed. Recently, similar enhancement effects on plant growth are observed between plasma activated water and artificially generated water through mixing with reactive species (Jirešova et al., 2022). This demonstrates the importance of reactive species in plasma activated water. However, synergistic effects of various species, effects of secondary species resulted from reactions between species, or other physical effects, which can be generated mostly by plasma activated water or solution, should be also considered.

## 5 Conclusion

Global population growth and climate change have compelled researchers to develop new crop production methods to fulfill demand while being environmentally friendly. Indoor agriculture and hydroponic culture have drawn increasing attention from researchers as alternative solutions. Our study shows that using plasma gas could greatly enhance the fertility of nutrient solutions for plant growth in hydroponic culture systems. In cultures grown in plasma gas-treated nutrient solution, bok choy showed increased physiological and biochemical activity, plant growth, and tolerance to salinity stress. Therefore, our study provides an additional experimental basis for plasma technology as a potential tool to be integrated with hydroponic plant culture to boost plant production, which is essential in meeting the increasing demands of the global population. We suggest that H_2_O_2_, a vital reactive oxygen species involved in various physiological processes and produced in plasma gas-treated nutrient solutions, may have acted as a signaling molecule, playing a crucial role in stimulating plant growth. The increase in intracellular NO in plant root cell division and elongation areas seems to be related to this, but further investigation is needed in relation to topic. Furthermore, it cannot be excluded that besides H_2_O_2_, various reactive species and secondary species resulted from reactions between species can be generated in plasma gas treated Hoagland solution, and mixture of these species can produce the synergistic and intensive effects on plant cells, which can affect plant growth and development. This can be one of advantages of plasma treated solution compared to other chemical solutions as a promising tool for applying to hydroponic agriculture practices. However, intensive chemical analysis on plasma treated solution is a pre-requirement for elucidating the mechanistic basis of plasma mediated plant hydroponic culture.

## Data Availability

The original contributions presented in the study are included in the article/**Supplementary Material**. Further inquiries can be directed to the corresponding authors.
