# Supplementary material for: Non-thermal plasma enhances growth and salinity tolerance of bok choy (Brassica rapa subsp. chinensis) in hydroponic culture
Source: Front Plant Sci. 2024 Sep 23;15:1445791. doi: 10.3389/fpls.2024.1445791 (PMC11456478; doi:10.3389/fpls.2024.1445791)
Supplement: Supplementary file 1 [file DataSheet1.docx]

Supplementary Material

Non-thermal plasma enhances growth and salinity tolerance of bok choy (*Brassica rapa* subsp. *chinensis*) in hydroponic culture

Mayura Veerana^1,*,†^, Wirinthip Ketya^2,†^, Eun-Ha Choi^2,3^, Gyungsoon Park^2,3,*^

^1^Department of Applied Radiation and Isotopes, Faculty of Science, Kasetsart University, Bangkok, Thailand

^2^Plasma Bioscience Research Center, Department of Plasma-Bio Display, Kwangwoon University, Seoul, Republic of Korea

^3^Department of Electrical and Biological Physics, Kwangwoon University, Seoul, Republic of Korea

**^*^Correspondence:** Mayura Veerana: fscimuv@ku.ac.th, Gyungsoon Park: gyungp@kw.ac.kr

**^†^**These authors contributed equally to this work.

# Supplementary Figures

(a)


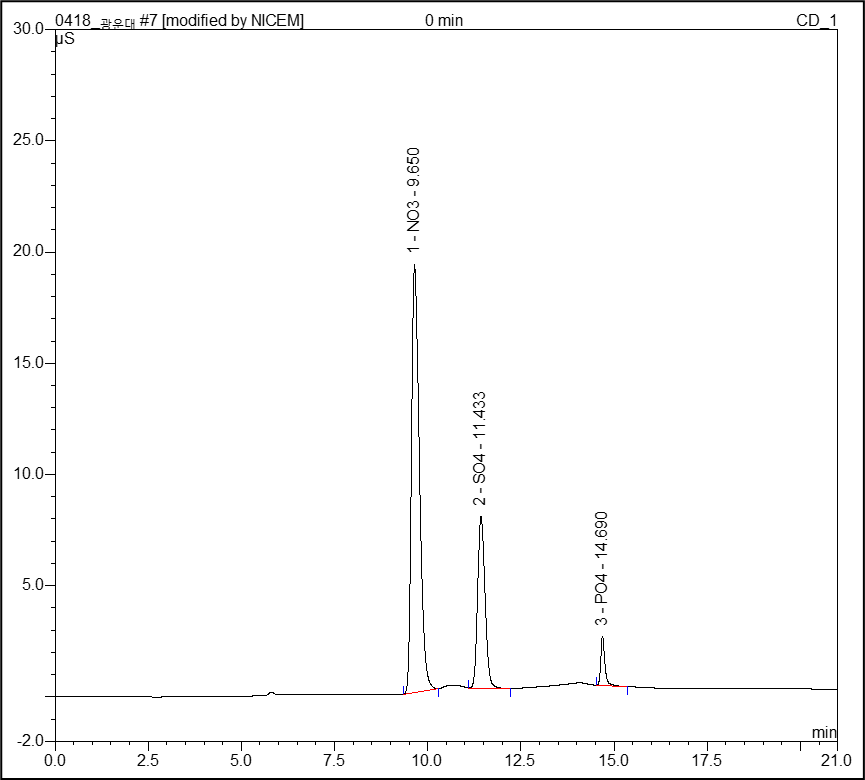

(b)


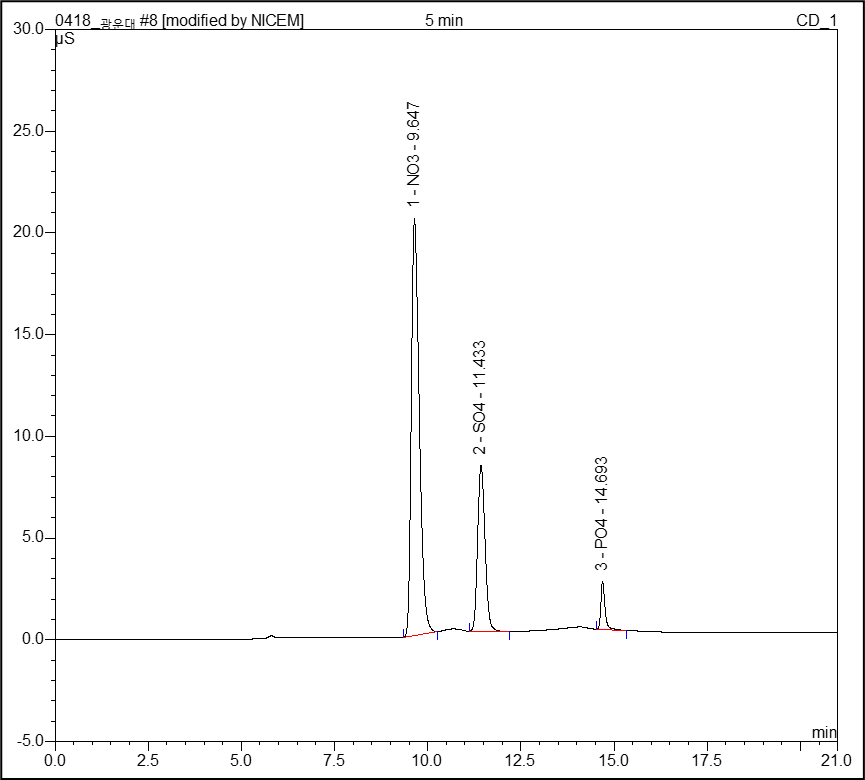

(c)


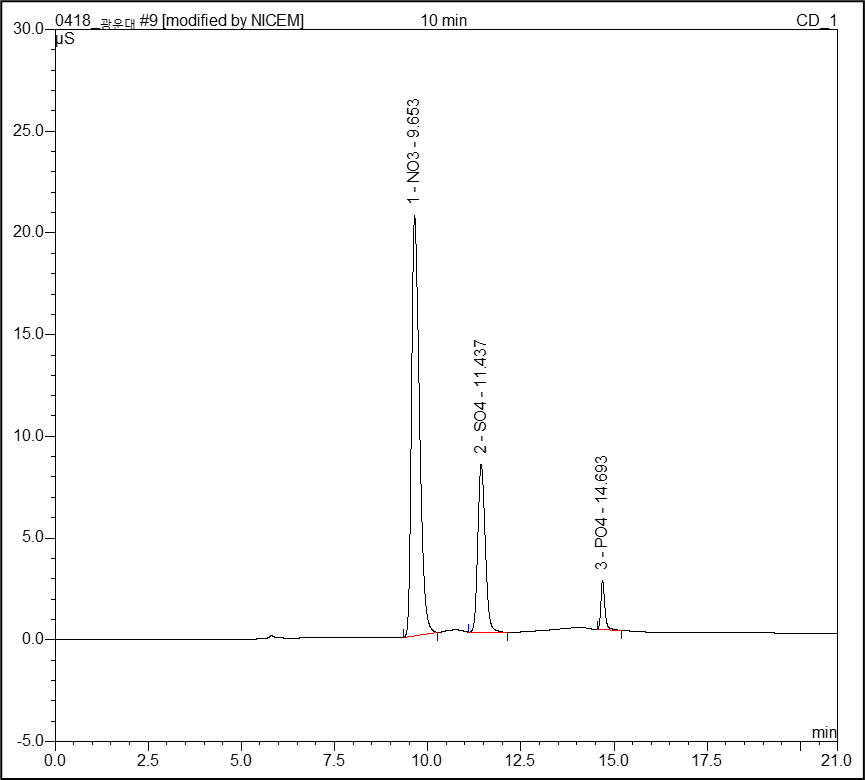

**Supplementary Figure S1.** Results of ion chromatography analysis. Anions in Hoagland solution were analyzed after plasma gas treatment for 0 min (a), 5 min (b), and 10 min (c).


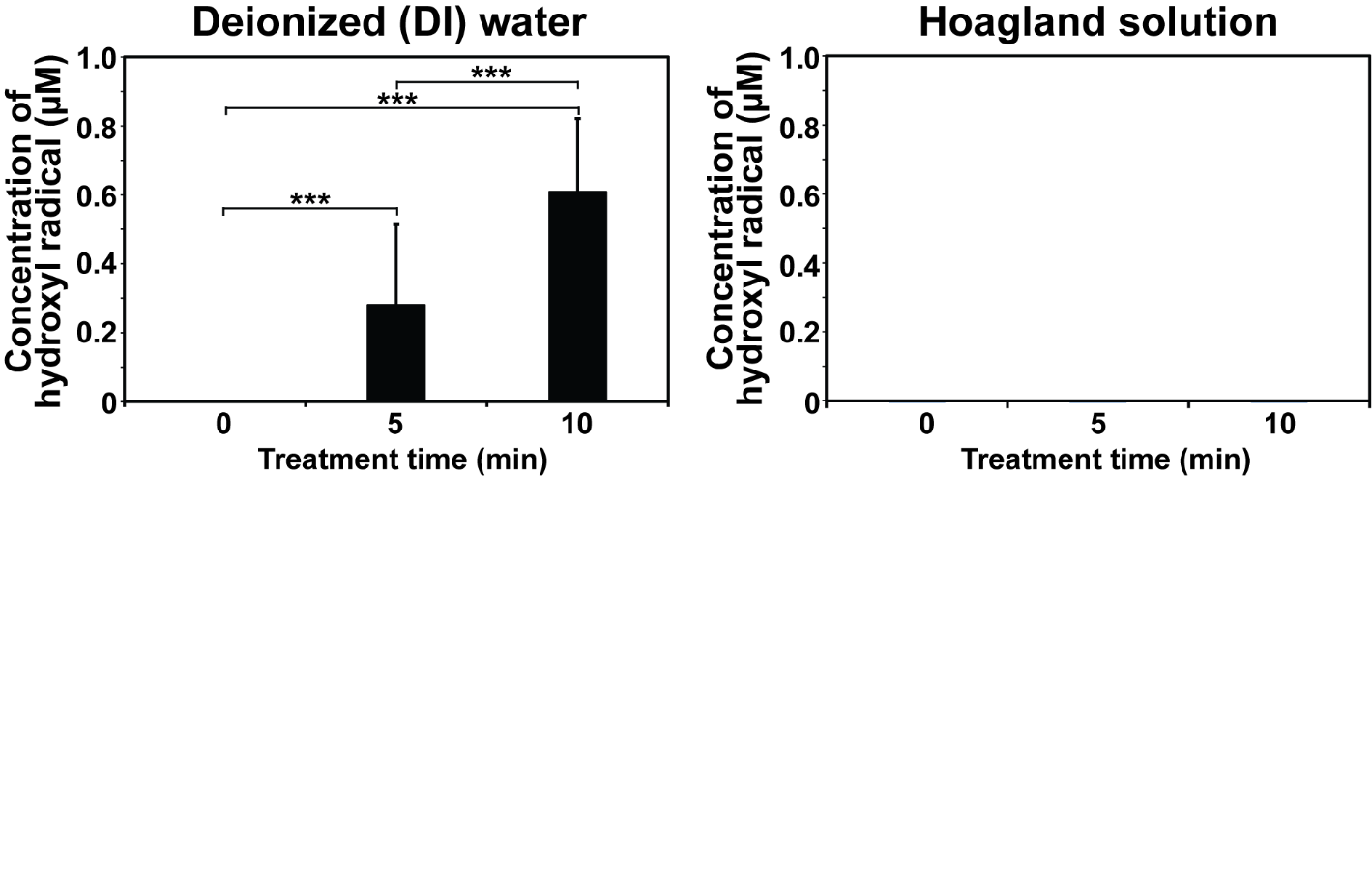


**Supplementary Figure S2.** Level of hydroxyl radical in deionized water (left graph) and Hoagland solution (right graph) injected with plasma gas for 0, 5, and 10 min. Each value represents the mean and standard deviations of replicate measurements; n = 6 or n = 9. ****p* < 0.001.

##
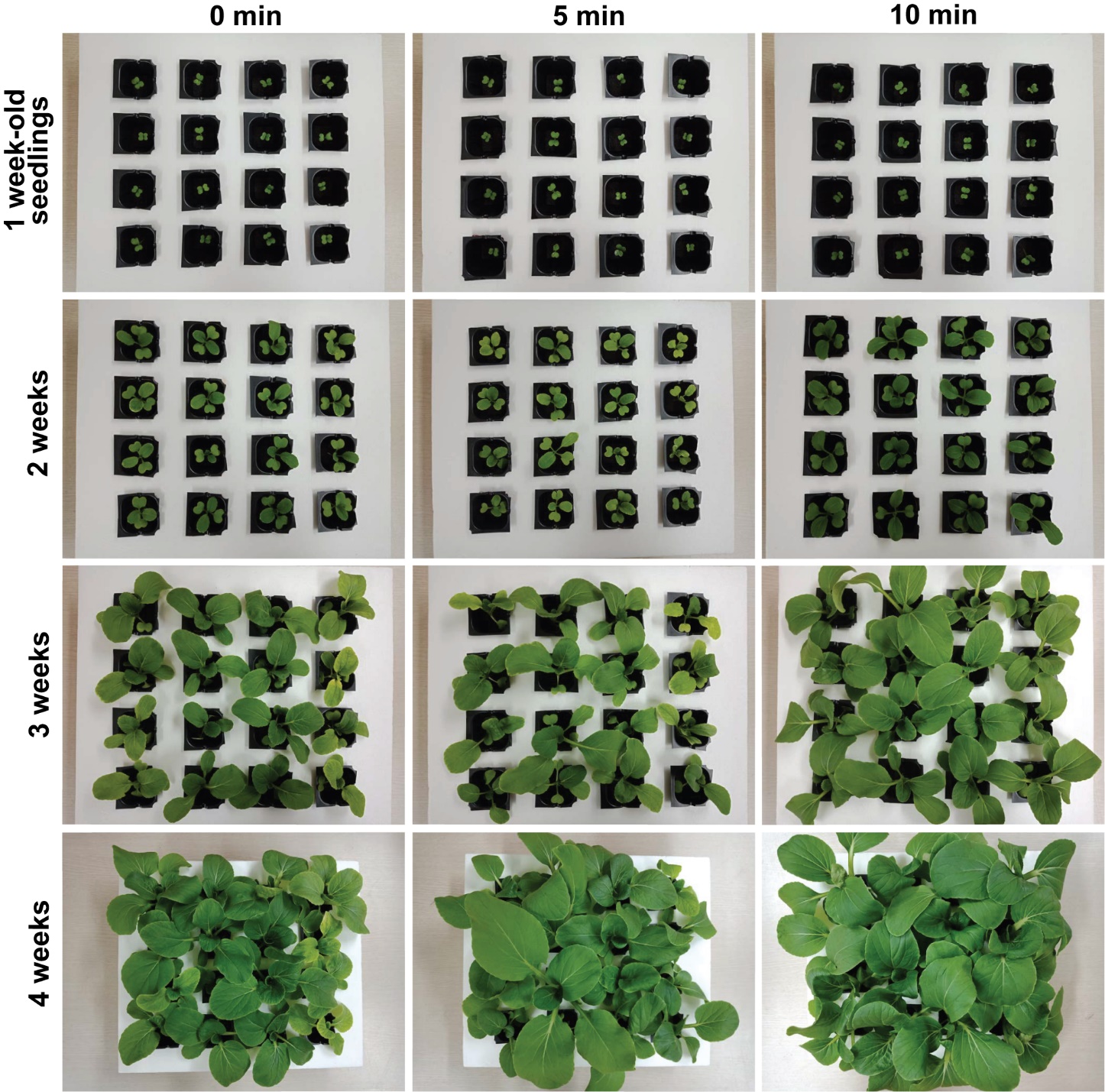


**Supplementary Figure S3.** Photograph of bok choy plants grown in Hoagland solution injected with plasma gas for 0, 5, and 10 min once a week.


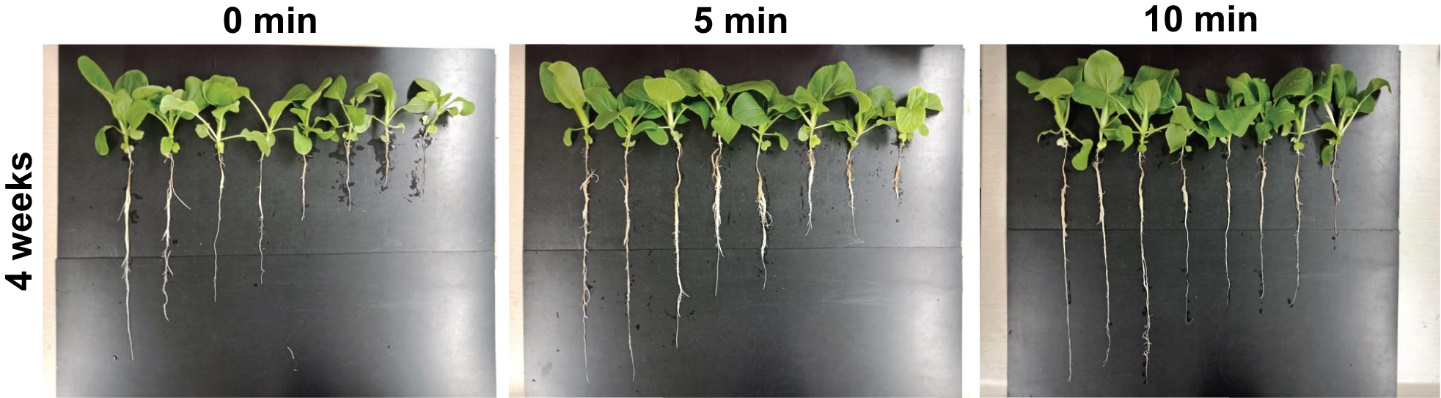


**Supplementary Figure S4.** Photograph of harvested bok choy plants grown for 4 weeks in Hoagland solution injected with plasma gas for 0, 5, and 10 min once a week.


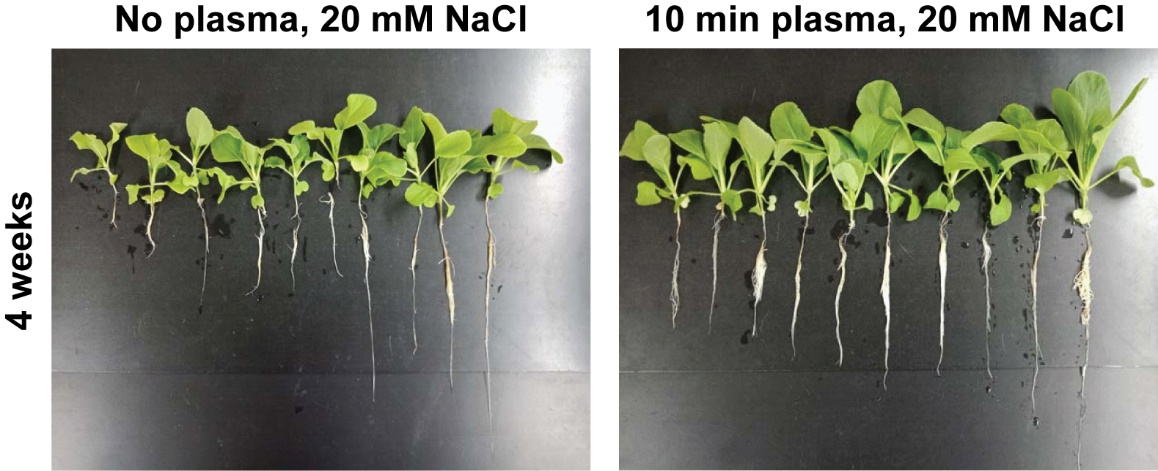


**Supplementary Figure S5.** Photograph of harvested bok choy plants grown for 4 weeks in Hoagland solution containing 20 mM NaCl injected with plasma gas for 0 and 10 min once a week.


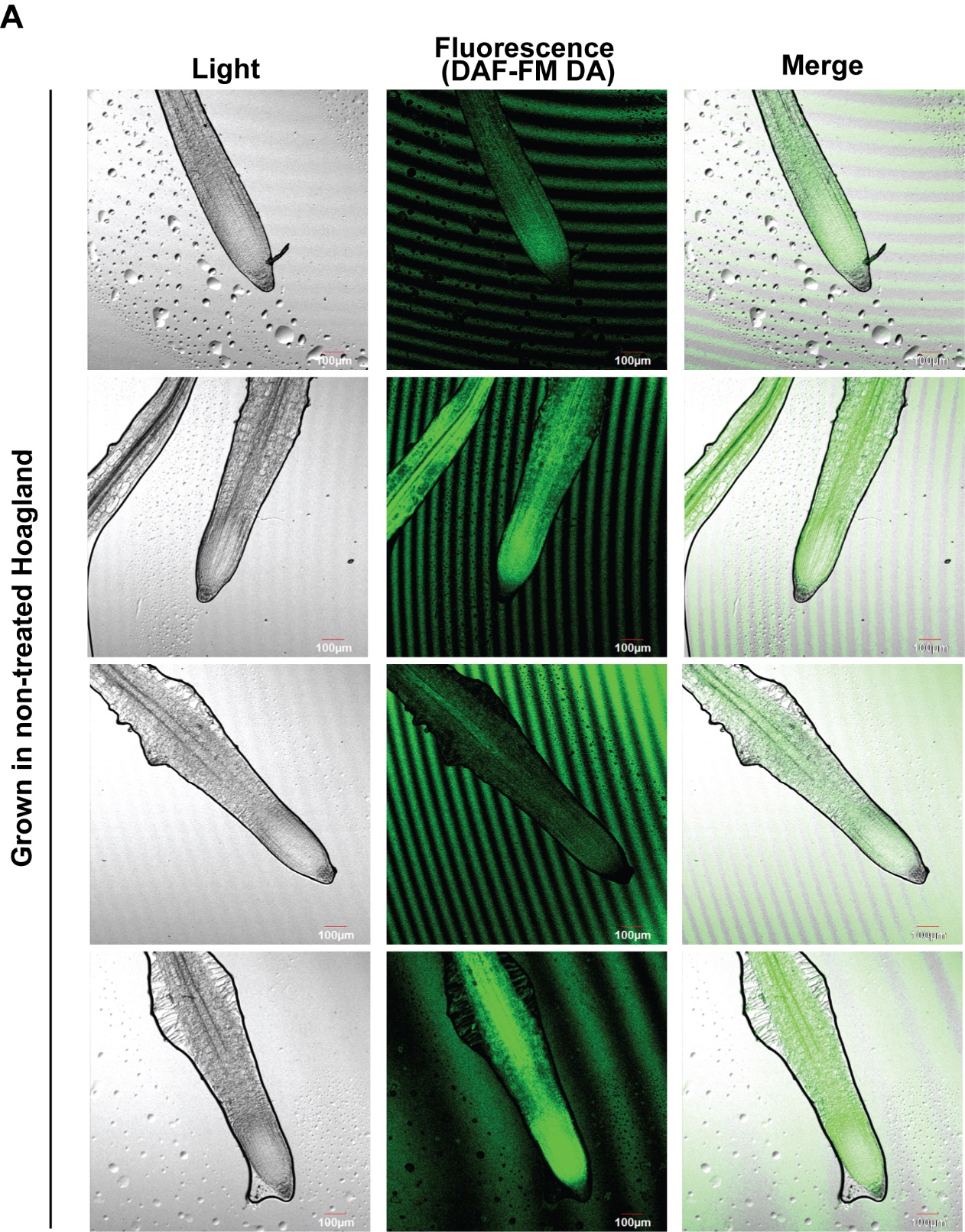


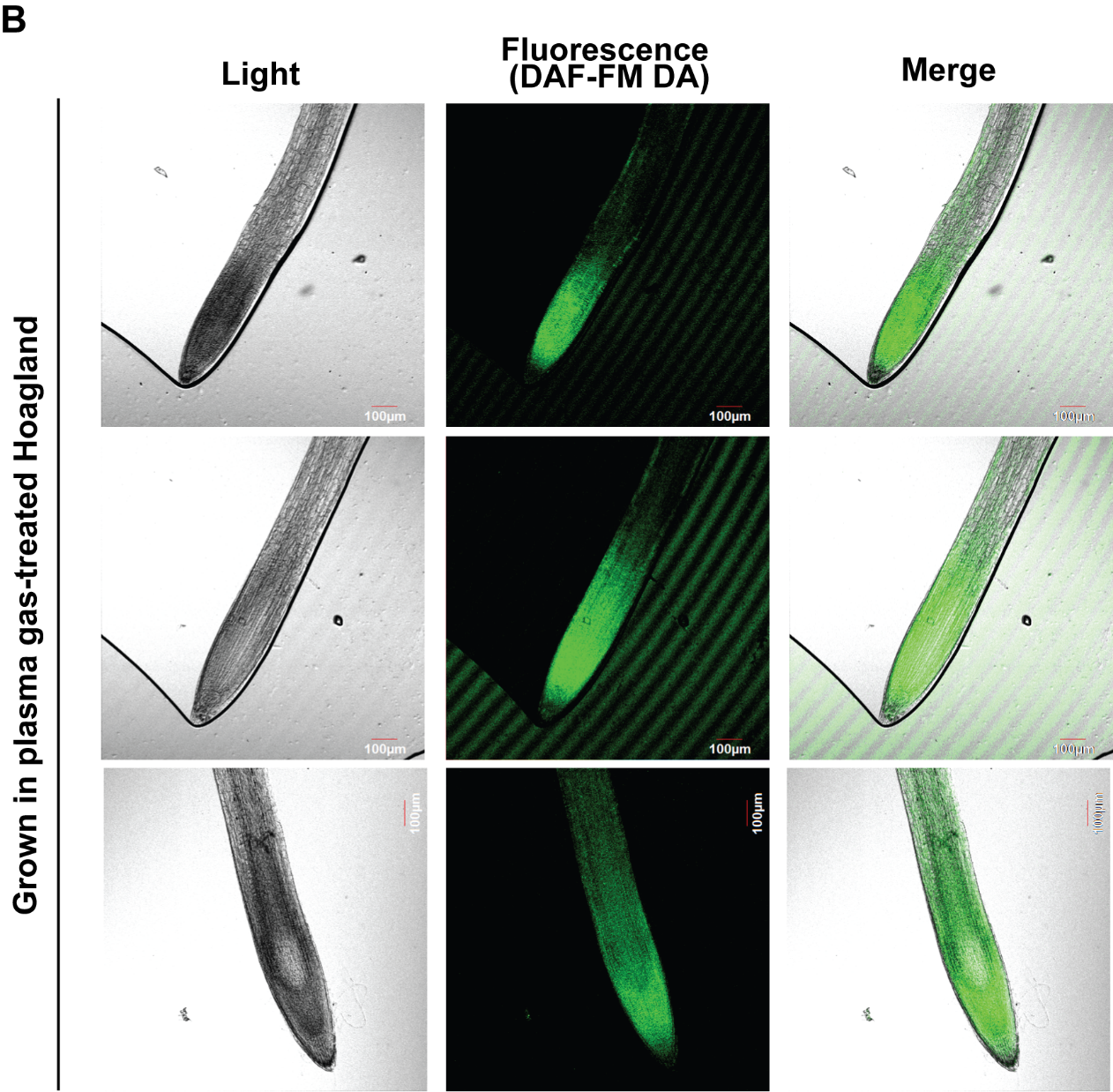


**Supplementary Figure S6.** Intracellular NO labeled with DAF-FM DA in roots of bok choy plants grown in Hoagland solution treated with plasma gas for 0 min (A) and 10 min (B).
